# Berberine Protects Human Retinal Pigment Epithelial Cells from Hydrogen Peroxide-Induced Oxidative Damage through Activation of AMPK

**DOI:** 10.3390/ijms19061736

**Published:** 2018-06-12

**Authors:** Shuai Li, Uma Gaur, Cheong-Meng Chong, Shaofen Lin, Jiankang Fang, Zhiwen Zeng, Haitao Wang, Wenhua Zheng

**Affiliations:** 1 Faculty of Health Science, University of Macau, Taipa, Macau 999078, China; yb67619@umac.mo (S.L.); gaur.uma2906@gmail.com (U.G.); legendhero91@gmail.com (C.-M.C.); yb57646@umac.mo (J.F.); zengzw1122@gmail.com (Z.Z.); 2 State Key Laboratory of Ophthalmology, Zhongshan Ophthalmic Center and School of Pharmaceutical Sciences, Sun Yat-sen University, Guangzhou 510000, China; linshaofen1970@163.com; 3 Department of Neuropharmacology and Novel Drug Discovery, School of Pharmaceutical Sciences, Southern Medical University, Guangzhou 510000, China; wht821@smu.edu.cn; 4 UM Zhuhai Research Institute, Zhuhai 519000, China

**Keywords:** berberine, age-related macular degeneration, D407 cells, AMPK

## Abstract

Age-related macular degeneration (AMD) is the leading cause of central vision loss in the elderly with less effective treatment, especially for dry AMD (90% of AMD). Although the etiology of this disease is not well elucidated, increasing evidences indicate that excessive reactive oxygen species (ROS) impairing the physiological functions of retinal pigment epithelium (RPE) cells may be one of the main causes. Therefore, it could be a great strategy to find some drugs that can effectively protect RPE cells from oxidative damage which is desired to treat and slow the process of AMD. In the present study, a well-known traditional Chinese medicine berberine (BBR) was found to suppress hydrogen peroxide (H_2_O_2_)-induced oxidative damage in D407 cells, a human RPE cell line. Pre-treatment of D407 cells with BBR significantly suppressed H_2_O_2_-induced cell apoptosis by restoring abnormal changes in nuclear morphology, preventing the decline of mitochondrial membrane potential, reducing lactate dehydrogenase release and inhibiting caspase 3/7 activities induced by H_2_O_2_. Western blot analysis showed that BBR was able to stimulate the phosphorylation/activation of AMPK in a time- and dose-dependent manner in D407 cells, while treatment of cells with AMPK pathway inhibitor Compound C, or knockdown of the AMPK by specific siRNA blocked the effect of BBR. Similar results were obtained in primary cultured human RPE cells. Taken together, these results demonstrated that BBR was able to protect RPE cells against oxidative stress via the activation of AMPK pathway. Our findings also indicate the potential application of BBR in AMD treatment.

## 1. Introduction

One of the most devastating retinal disease is age-related macular degeneration (AMD), which is also the major cause of blindness throughout the world. The prominent characteristic of AMD is the loss of central vision due to the progressive degeneration of the macula [1,2,3]. The number of AMD patients is predicted to increase two-fold in the coming decades due to an increasingly aged population [4]. The disease can be divided into dry and wet AMD forms and the dry form of the disease is more prevalent accounting for up to 90% of all cases [5]. Continued intraocular injections are the current treatment strategy to prevent progression of wet AMD (10%) [6]. However, the dry AMD still lacks efficient treatment and calls for the development of effective therapies. Although the mechanisms behind the origin and development of AMD are not fully understood, increasing studies have indicated that chronic inflammation, oxidative stress, and apoptosis might have an important role in the development of dry AMD [7,8]. Therefore, discovery of effective candidate drugs which can prevent or delay these injuries are urgently required to treat or slow down the process of dry AMD.

The layer of retinal pigment epithelial (RPE) cells in the macula has been reported to break down or get thin in the dry AMD [9]. RPE is a pigmented monolayer which plays crucial roles in retinal functions [10]. The degeneration of RPE with advancing age causes the death of photoreceptor cells thereby leading to vision loss [11,12,13]. The retina has been shown to have the highest oxygen consumption in comparison to other tissues, which indicates that the retinal RPE cells are more prone to oxidative stress [14,15]. Oxidative damage occurs while reactive oxygen species (ROS) causes lipid peroxidation, oxidation of enzymes, and DNA breakage resulting in irreversible damage to the cells. Increasing studies have suggested that oxidative stress induced by chemical oxidants such as hydrogen peroxide (H_2_O_2_) leads to RPE damage and death [16,17,18]. These findings provide experimental evidences in support of oxidative stress playing a crucial role in RPE degeneration. Some studies have shown that use of additional antioxidants to suppress oxidative stress can avoid oxidative damage and maintain the function of retina [19,20,21].Therefore, suppressing oxidative stress-induced RPE cell injury may be a potential strategy for delaying the progression of dry AMD.

**Figure 1:**
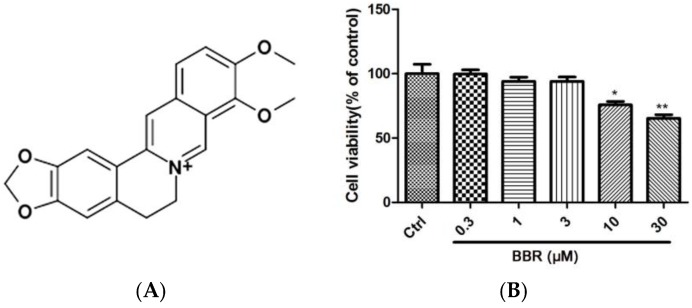

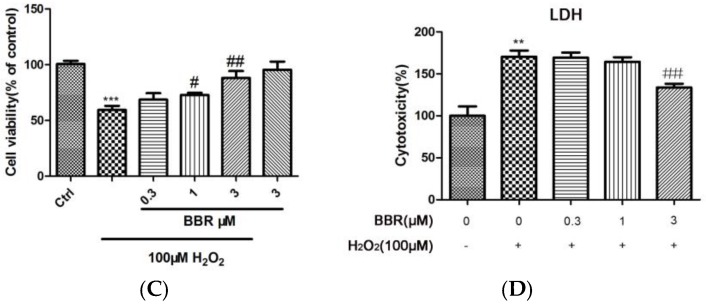
Protective effects of berberine (BBR) against H_2_O_2_-induced cytotoxicity in D407 cells. (**A**) The structure of BBR; (**B**) D407 cells were treated with BBR (0.3 to 30 µM) or 0.1% dimethyl sulfoxide (DMSO) (vehicle control) for 24 h and cell viability was measured using 3-(4,5-dimethylthiazol-2-yl)-2,5-diphenyl tetrazolium bromide (MTT) assay. Cells were pre-treated with BBR at indicated concentration or 0.1% DMSO (vehicle control) for 2 h and then incubated with or without 100 µM H_2_O_2_ for further 24 h. Cell viability and the release of lactate dehydrogenase (LDH) were measured by MTT assay (**C**) and LDH assay (**D**), respectively. * indicates *p* < 0.05, ** indicates < 0.01, *** indicates *p* < 0.001 versus the control group; ^#^ indicates *p* < 0.05, ^##^ indicates *p* < 0.01 versus the H_2_O_2_-treated group were considered significantly different.

Previously, we successfully created an H_2_O_2_-induced RPE cell injury model with D407 cells to screen potential protectants from a library of active components of various Chinese medicines [22]. Berberine (BBR, the structure of BBR is shown in Figure 1A), is a natural alkaloid compound derived from *Coptis chinensis,* is found to efficiently increase the D407 cells’ viability from the damage caused by H_2_O_2_ exposure between various Chinese medicines in our lab. For decades, BBR has been widely used in China as a medication for diarrhea. Various clinical studies conducted in the recent years have shown its therapeutic potential in many types of chronic diseases [23]. Accumulated studies suggested that BBR is endowed with several pharmacological activities, including anti-tumor activity [24], cardiovascular-protective actions [25,26], anti-inflammatory effects [27] and it has also been found to inhibit the expression of inflammatory cytokines in ARPE-19 cells cultured in the presence of TNF-α [28]. In addition, BBR also exhibited various other biological effects such as glucose regulation and lipid metabolism in vitro and in vivo [29,30]. However, whether BBR exerts any protective effects against H_2_O_2_ insult in RPE cells and the underlying mechanisms are still unknown.

In this study, we found that the protective effects of BBR against H_2_O_2_-induced oxidative damage in D407 RPE cells and primary cultured hRPE cells were executed via restoring the abnormal changes in nuclear morphology, intracellular ROS, mitochondrial membrane potential, and caspase activation. We also demonstrated that the protective effect of BBR is mediated via the AMPK pathway. These findings suggested that BBR administration might be considered as a potential therapeutic approach for the treatment of AMD.

## 2. Results

### 2.1. BBR Reduced H_2_O_2_-Induced D407 Cell Death

D407 cells were incubated with different concentrations of BBR for 24 h, in order to evaluate the cytotoxicity of BBR, and cell viability was assessed using 3-(4,5-dimethylthiazol-2-yl)-2,5-diphenyl tetrazolium bromide (MTT) assay. As shown in Figure 1B, BBR with a concentration from 0.3 to 3 μM did not cause any cytotoxicity in D407 cells compared to the control group. Therefore, these concentrations of BBR were chosen in further experiments. To investigate the protective effects of BBR on H_2_O_2_-induced D407 cell death, D407 cells were treated with BBR for 2 h before being exposed to H_2_O_2_ for 24 h. The result from MTT assay showed that treatment of 100 μM H_2_O_2_ resulted in a significant reduction of cell viability, whereas pre-treatment with 1 or 3 μM BBR significantly attenuated H_2_O_2_-induced cell viability loss in a concentration-dependent manner (Figure 1C). The protective activity of BBR was also confirmed by the lactate dehydrogenase (LDH) assay as shown in Figure 1D, in which pre-treatment with 3 μM BBR for 2 h significantly reduced H_2_O_2_-induced LDH leakage.

### 2.2. BBR Attenuated H_2_O_2_-Induced Apoptosis in D407 Cells

**Figure 2:**
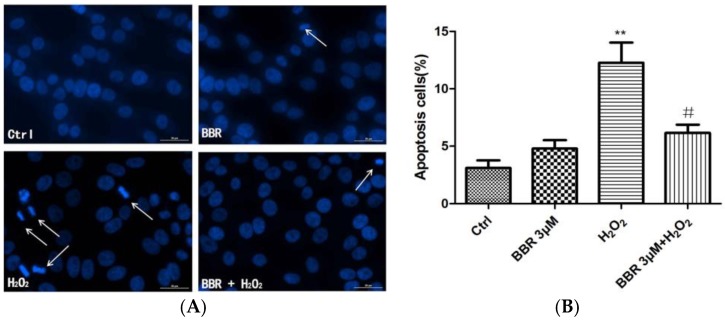
BBR attenuated H_2_O_2_-induced apoptosis in D407 cells. Apoptosis of D407 cells was detected by Hoechst 33342 staining and visualized by fluorescence microscopy. The number of apoptotic nuclei with condensed chromatin was counted from the photomicrographs and presented as a percentage of the total number of nuclei. ** indicates *p* < 0.01 versus the control group; ^#^ indicates *p* < 0.05 versus the H_2_O_2_-treated group were considered significantly different.

D407 cells pretreated with BBR were further exposed to 100 μM H_2_O_2_ for 24 h, and stained with Hoechst 33342.The results showed that 100 μM H_2_O_2_ caused remarkable nuclei condensation in cells. However, these changes induced by H_2_O_2_ were not seen when the cells were pre-treated with 3 μM BBR (Figure 2). BBR itself did not lead to nuclear morphological changes in D407 cells.

### 2.3. BBR Attenuated the Loss of Mitochondrial Membrane Potential and the Activation of Caspase 3/7 Induced by H_2_O_2_

**Figure 3:**
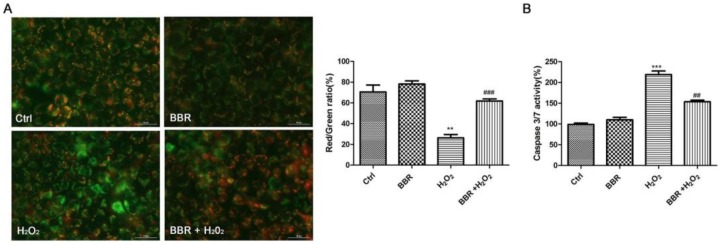
BBR attenuated H_2_O_2_-induced loss of mitochondrial membrane potential (Δψm) and activation of caspase 3/7 in D407 cells. After pre-treatment with 3 μM BBR or 0.1% DMSO (vehicle control) for 2 h, D407 cells were incubated with or without 100 μM H_2_O_2_ for another 24 h. (**A**) Δψm was determined by JC-1 assay; (**B**) Quantification of caspase 3/7 activity was determined by caspase 3/7 activity assay. ** indicate *p* < 0.01, *** indicate *p* < 0.001 versus the control group; ^##^ indicate *p* < 0.01, ^###^ indicate *p* < 0.001 versus the H_2_O_2_-treated group were considered significantly different.

The dysregulated mitochondrial functioning causes the loss of mitochondrial membrane potential (Δψm). To find out whether BBR could bring down the H_2_O_2_-induced Δψm loss, the mitochondrial membrane potential was determined by analyzing the red/green fluorescent intensity ratio of JC-1 staining in D407 cells. Exposure of D407 cells to 100 μM H_2_O_2_ resulted in an increase in green fluorescence intensity which indicated that mitochondrial membrane potential is dissipated (Figure 3A). Pre-treatment with BBR at 3 μM concentration for 2 h significantly attenuated H_2_O_2_-induced Δψm loss. This indicated that H_2_O_2_ significantly decreased the mitochondrial membrane potential, an effect which was remarkably brought down when cells were pre-treated with BBR. To further verify the protective effects of BBR, Caspase 3/7 activity was determined. Caspase 3/7 is a main biomarker of the cell apoptosis. Treatment of D407 cells with H_2_O_2_ (100 μM) for 24 h resulted in an increase in caspase 3/7 activity by more than two-fold in comparison to the control group as shown in Figure 3B. In contrast, pre-treatment with BBR significantly brought down the caspase 3/7 activation initiated by H_2_O_2._

### 2.4. BBR Attenuated the H_2_O_2_-Induced ROS Production

**Figure 4:**
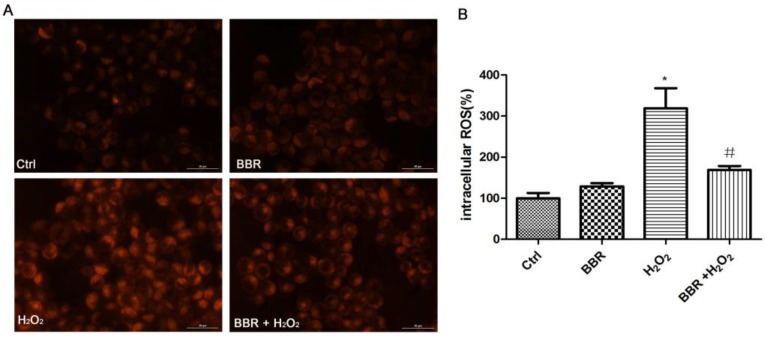
BBR attenuated the H_2_O_2_-induced reactive oxygen species (ROS) production. Exposure of D407 cells to 100 μM H_2_O_2_ resulted in an increase of ROS level. Pre-treatment of cells with BBR at 3 μM inhibited the H_2_O_2_-induced increase of ROS level in D407 cells. After pre-treatment with 3 μM BBR or 0.1% DMSO (vehicle control) for 2 h, D407 cells were incubated with or without 100 μM H_2_O_2_ for another 24 h. * indicates *p* < 0.05 versus the control group were considered statistically different. ^#^ indicates *p* < 0.05 versus the H_2_O_2_-treated group were considered statistically different.

The cytotoxicity of H_2_O_2_ was mediated by increased levels of ROS in the D407 cell. Therefore, we used CellROX Deep Red Reagent staining to investigate the effects of BBR on the oxidation induced by H_2_O_2_. As shown in Figure 4, exposure of D407 cells to 100 μM H_2_O_2_ resulted in an increase of ROS level. However, pre-treatment of cells with BBR at 3μM inhibited the H_2_O_2_-induced increase of ROS level in D407 cells.

### 2.5. BBR Stimulated AMPK Phosphorylation in D407 Cells

**Figure 5:**
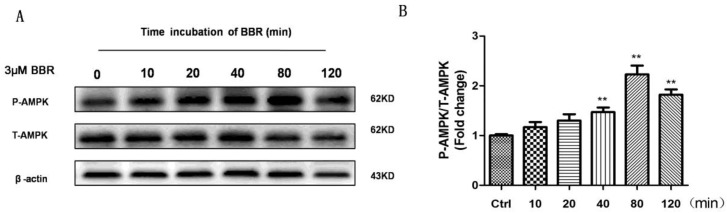

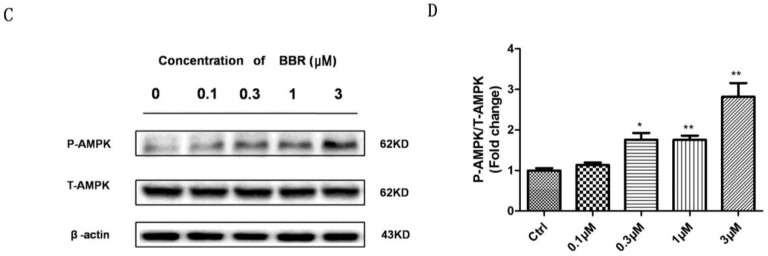
BBR stimulated AMPK phosphorylation in D407 cells. (**A**) D407 cells were treated with 3 µM BBR for various time points as indicated in the Figure and the phosphorylation of AMPK (P-AMPK), total AMPK (T-AMPK), and β-actin were detected by Western blotting with specific antibodies; (**B**) Quantification of representative protein band from Western blotting. * *p* < 0.05 versus the control group was considered significantly different; (**C**) D407 cells were treated with various concentrations of BBR for 80 min and the expression of phosphorylated AMPK (P-AMPK), total AMPK (T-AMPK), and β-actin were detected by Western blotting with specific antibodies (**D**). Quantification of representative protein band from Western blotting. * indicate *p* < 0.05, ** indicate *p* < 0.01 versus the control group was considered significantly different.

BBR has been shown to activate AMPK signaling in many cell types [31,32,33,34]. In order to find out whether AMPK signaling is regulated by BBR in D407 cells, the cells were treated with 3 μM BBR for different time points or with different concentrations of BBR for 80 min as shown in Figure 5, and the phosphorylation level of AMPK was checked by Western blotting. As shown in Figure 5A,B, BBR time-dependently stimulated the phosphorylation of AMPK. The stimulation was seen at 10 min, reached maximum at 80 min and decreased afterwards. Figure 5C,D showed the concentration-dependent stimulation of phosphorylation of AMPK by BBR. The lowest effective concentration of BBR found to stimulate AMPK phosphorylation was 0.3 μM and the maximum effect was observed at 3 μM.

### 2.6. The Protective Effect of BBR Was Mediated by AMPK Signaling

**Figure 6:**
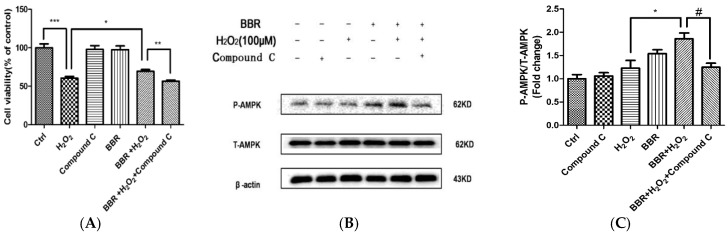
AMPK specific inhibitor Compound C blocked the protective effect of BBR in D407 cells. (**A**) D407 cells were pre-treated with 5 µM Compound C for 30 min and 3 µM BBR for 2 h and then incubated with or without H_2_O_2_ for a further 24 h. Cell viability was measured by MTT assay. * indicates *p* < 0.05. ** indicates *p* < 0.01, *** indicates *p* < 0.001 was considered significantly different; (**B**) D407 cells were pre-treated with 5 µM Compound C for 30 min and 3 µM BBR for 80 min and then incubated with or without H_2_O_2_ for a further 2 h. The expression of phosphorylated AMPK, total AMPK, and β-actin were detected by Western blotting with specific antibodies; (**C**) Quantification of representative protein band from Western blotting. * indicates *p* < 0.05versus the H_2_O_2_ group was considered significantly different. ^#^ indicates *p* < 0.05 versus the BBR + H_2_O_2_-treated group was considered significantly different.

**Figure 7:**
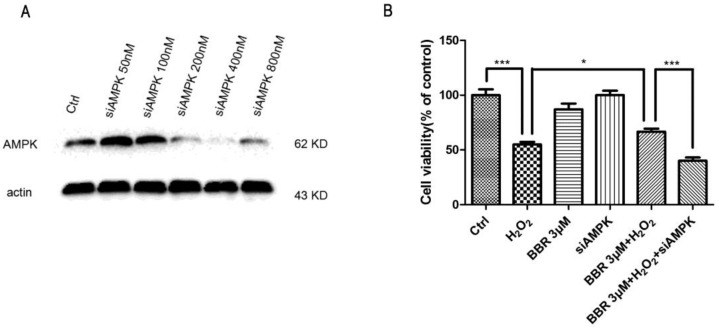
Knocking down the expression of AMPK by siRNA blocked the protective effect of BBR in D407 cells. (**A**) Cells were transfected with different concentrations of siAMPK for 48 h and the expression of AMPK or β-actin was detected by Western blotting; (**B**) Cells were transfected with 400 nM siAMPK, transfected cells were seeded in 96-well, 24 h later treated with 3 µM BBR or 0.1% DMSO (vehicle control) for 2 h and then incubated with or without 100 µM H_2_O_2_ for another 24 h. Cell viability were measured by MTT assay. * indicates *p* < 0.05, *** indicates *p* < 0.001 was considered significantly different.

To further confirm the involvement of AMPK signaling in the protective effects of BBR, AMPK specific inhibitor Compound C was introduced to the following experiment. As shown in Figure 6A, the result of MTT assay indicated that the protective effect of BBR was abolished by pre-incubation with Compound C, suggesting that AMPK signaling activation is essential in the protective effects of BBR. In line with this finding, Western blotting also showed that Compound C, at the concentration that blocked the protective effect of BBR, also blocked BBR-induced AMPK phosphorylation (Figure 6B). To further ascertain the mediated effect of AMPK, we used specific siRNA for AMPK to knock down the expression of AMPK in D407 cells. As expected, down regulation of AMPK (Figure 7A) notably reduced the protective effect of BBR in D407 cells (Figure 7B).

### 2.7. BBR Protected Primary Cultured Human RPE Cells against H_2_O_2_ Induced Injury via the AMPK Pathway

**Figure 8:**
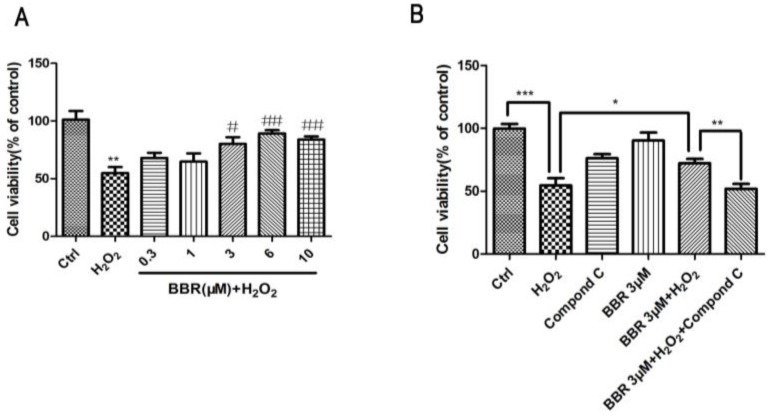
BBR protected primary cultured human RPE cells against H_2_O_2_ induced injury via the AMPK pathway. (**A**) Primary cultured hRPE cells were pre-treated with different concentrations of BBR for 2 h, then incubated with or without H_2_O_2_ for a further 24 h. Cell viability was measured by MTT assay. ** indicates *p* < 0.01 versus the control group was considered significantly different, ^#^ indicates *p* < 0.05, ^##^ indicates *p* < 0.01 versus H_2_O_2_ group was considered significantly different; (**B**) Primary cultured hRPE cells were pre-treated with 5 µM Compound C for 30 min and 3 µM BBR for 2 h and then incubated with or without H_2_O_2_ for further 24 h, and cell viability was measured using the MTT assay. * indicates *p* < 0.05, ** indicates *p* < 0.01, *** indicates *p* < 0.001 was considered significantly different.

To verify whether the protective effect of BBR is limited to D407 cell line, we also characterized its protective effects on primary cultured human retinal pigment epithelial cells (hRPE cells). The effect of BBR on the primary cultured hRPE cells exposed to H_2_O_2_ injury is presented in Figure 8. BBR successfully conferred protection of primary cultured hRPE cells against H_2_O_2_ insult in a concentration-dependent manner. Moreover, the protective effect of BBR is also inhibited by Compound C in these cells. These results are in accordance with the results from D407 cells, which further confirmed that BBR is able to protect hRPE cells from oxidative stress via the AMPK pathway.

## 3. Discussion

Clinical and experimental data supports that chronic oxidative stress is a primary contributing factor to numerous retinal degenerative diseases, such as AMD [35]. The clinical eye samples obtained from dead patients have shown pervasive free radical impairment in the DNA, lipids, proteins and mitochondria of RPE cells [36]. Also, several mouse models of chronic oxidative stress have been shown to develop many of the pathological signs of AMD [37,38]. More importantly, data from animal and clinical studies suggested that the use of antioxidant drugs could be a potential strategy for delaying the progression of AMD and vision loss [39,40,41]. BBR, a well-known constituent of the Chinese herb Huanglian has displayed antioxidant properties in a variety of cells [27,42,43,44,45]. Recently, it was reported that systemic BBR was able to protect against light-induced retinal degeneration associated with diminished oxidative stress in the retina [46]. In the present study, we found that H_2_O_2_ (100 μM) exposure lead to the collapse of the Δψm and increase of ROS in RPE cells, while pre-treatment of BBR significantly reduced these abnormal changes in D407 RPE cells. During treatment with H_2_O_2_, cells are exposed to high concentrations of ROS, which consequently disrupts the balance between oxidants and antioxidants, resulting in apoptosis and/or necrosis of cells. In the current study, the frequent type of cell death observed in H_2_O_2_-treated RPE cells was apoptosis, which was significantly reduced by BBR. Furthermore, pretreatment with BBR prior to incubation with H_2_O_2_ significantlyattenuated the alteration caused by H_2_O_2_ exposure, such as the loss of cell viability, the elevation of LDH release and nuclear morphological changes, suggesting that anti-oxidant activity of BBR contributes to its protective effects. The concentration range of BBR used in our experiments had no toxicity in D407 cells which indicated that BBR is a safe protectant.

Mitochondrial dysfunctions have been implicated in the pathophysiology of several age-related diseases including AMD [47]. Mitochondrial apoptotic pathway activation resulting from mitochondrial deficiency plays a crucial role in the pathogenesis of retinal diseases [48]. It has been reported that compounds which improve mitochondrial functions may show beneficial effects in preventing AMD [49,50]. BBR has been shown to exert protective effects in disease models of various diseases such as hepatic disease [51], obesity [52], and myocardial ischemia [53], etc., by restoring the dysregulated mitochondrial function. And BBR has been showed to attenuate ischemia/reperfusion injury by inhibiting endoplasmic reticulum and mitochondrial stress pathways [54]. Moreover, BBR is believed to interplay with various enzymes, receptors and iron channel that are involved in mitochondria functions [23].

Consistent with above report, our results also revealed that BBR reversed H_2_O_2_-induced ROS accumulation, △ψm loss and suppress the activation of caspase 3/7 in D407 cells caused by H_2_O_2_. These findings indicated mitochondria injury plays an important role in the protective effect of BBR. Thus our findings for the first time showed that BBR could increase mitochondrial membrane potential in D407 cells treated with H_2_O_2_.As mitochondria are dynamic organelles with the ability to fuse (fusion) and divide (fission), mitochondria-related parameters, such as mitochondrial biogenesis, ATP production, expression of complex I-IV proteins and the level of MnSOD are pivotal indicators showing the biological functions of mitochondria. All these potential parameters are deserved to be investigated in the future.

BBR is a very well-known activator of AMPK which also triggers beneficial metabolic activities in diabetic as well as insulin-resistant states [55]. Recent reports have suggested the involvement of AMPK in mediating cell survival or apoptosis under stress condition [56,57,58]. It is reported that short term treatment with BBR results in ATP depletion, which further leads to AMPK activation and mitochondrial fragmentation [59,60]. Further studies suggested that BBR was able to reduce mitochondrial dysfunction induced by high fat diet and hyperglycemia in skeletal muscle via AMPK activation [52,59]. More interestingly, AMPK signaling axis was also reported to play a crucial role in RPE cell apoptosis [61]. In present study, we observed that BBR treatment lead to a significant increase in AMPK phosphorylationin D407 cells. In addition, BBR triggered protective effects against cell viability lose and cell apoptosis was attenuated by specific pharmacological inhibitors of AMPK kinase (Compound C) or specific siRNA for AMPK. Therefore, these results provided mechanistic evidences to support the notion that BBR-regulated protective effects against H_2_O_2_-induced oxidative stress in D407 cells are implemented via AMPK activation. Consequently, this protective effect of BBR was reproduced and confirmed in primary cultured human RPE cells.

**Figure 9:**
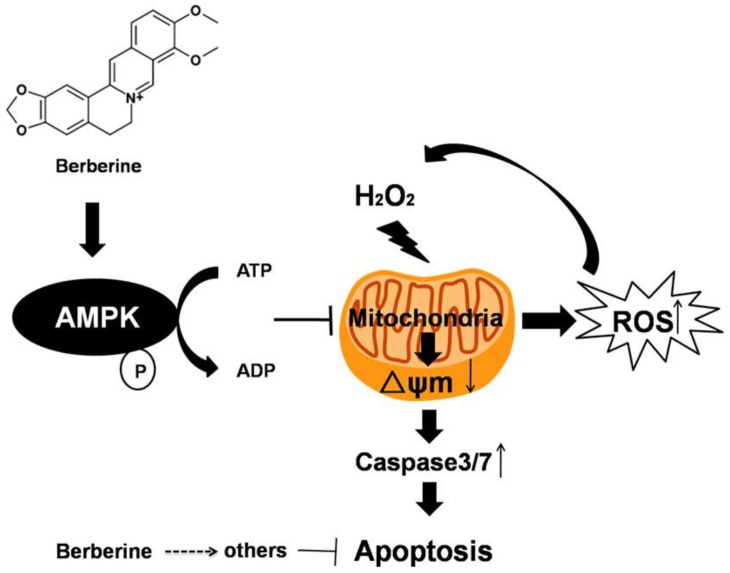
The possible mechanism of Berberine.

In summary, our findings demonstrated that BBR is able to significantly attenuate H_2_O_2_-induced oxidative injury in D407 cells through modulating Δψm and caspase 3/7 dependent pathway, inhibiting the generation of intracellular ROS, and activating AMPK signaling. Therefore, the protective activity of BBR to attenuate H_2_O_2_-mediated intrinsic mitochondrial apoptotic pathway are at least partially, mediated via the activation of AMPK signaling pathway (Figure 9). Our results offer support for the potential application of BBR in preventing or delaying oxidative stress–induced retinal pigment epithelial cell death in the process of dry AMD.

## 4. Materials and Methods

### 4.1. Materials

Human retinal pigment epithelial cell line D407 was obtained from cell bank, Sun Yat-sen University (Guangzhou, China); poly-l-lysine and bovine serum albumin were purchased from Sigma (St. Louis, MO, USA);dimethyl sulfoxide (DMSO). penicillin-streptomycin (PS); trypsin; Fetal bovine serum (FBS) and DMEM were purchased from Invitrogen (Carlsbad, CA, USA); methyl thiazolyl tetrazolium (MTT), JC-1, and Hoechst 33342 were purchased from Molecular Probes (Eugene, OR, USA). Caspase-Glo^®^ 3/7 Assay kit was purchased from Invitrogen, USA. CytoTox-ONE™ Homogeneous Membrane Integrity Assay (Promega, Madison, WI, USA) kit; CellROX Deep Red Reagent was purchased from Thermo Fisher Scientific, Waltham, MA, USA. Western blotting and protein quantification materials were purchased from Bio-Rad (Hercules, CA, USA). Super Signal West Pico chemiluminescent substrate was purchased from Thermo Scientific (Rockford, IL, USA). Anti-β-actin, phospho-AMPK and total AMPK antibodies were purchased from Cell Signaling Technology (Woburn, MA, USA). AMPK inhibitor (Compound C) was purchased from Selleckchem. siAMPK was purchased from Molecular Informatrix Laboratory.

### 4.2. Cell Culture

Cell line D407 was maintained and grown in DMEM culture medium with 10% FBS (heat-treated at 56 °C for 30 min). Streptomycin (100 μg/L) and penicillin (100 U/mL) were added into the medium. Cells were incubated at 37 °C with 5% CO_2_-humidified atmosphere. The medium was replaced every 3 days, and cells were sub-cultured by 0.25% trypsin treatment twice a week. The cells of passages 3 to 8 were used in further experiments. Primary Cultured Human retinal pigment epithelial (hRPE) cells were obtained from the State Key Laboratory of Ophthalmology, Zhongshan Ophthalmic Center and were approved by the Ethics Committee of Zhongshan Ophthalmic Center (2018KYPJ082, 15 May 2018). The cells were grown in DMEM/F12 culture medium augmented with 10% FBS and 1% penicillin/streptomycin in a humidified incubator at 37 °C and 5% CO_2_.

### 4.3. MTT Assay

Cell viability was checked by 3-(4,5-dimethylthiazol-2-yl)-2,5-diphenyl tetrazolium bromide (MTT) assay. In brief, D407 cells were grown in 96-well plates at a density of 5 × 10^4^ cells/mL. After serum deprivation, the D407 cells were exposed to reagents for 24 h. Then, MTT (0.5 mg/mL) was added into the plate and incubated for 3 h at 37 °C. The medium was aspirated from each well and DMSO (100 μL) was added to dissolve the formazan crystals. The data were quantified spectrophotometrically at wavelength of 490 nm.

### 4.4. LDH Assay

Through measuring the activity of lactate dehydrogenase (LDH) released into the incubation medium when cellular membranes are damaged, we can know the Cell cytotoxicity. D407 cells were seeded into 96-well plates (5 × 10^4^ cells/mL). The activity of LDH released in the medium was measured following the manufacturer’s instructions. Infinite M200 PRO Multimode Microplate reader was used to measure the fluorescent intensity at an excitation wavelength of 560 nm and emission wavelength of 590 nm.

### 4.5. Hoechst 33342 Staining

Apoptosis of cells was assessed by staining with the DNA binding dye Hoechst 33342. D407 cells were grown into 96-well plates (1 × 10^4^ cells/well). After suitable treatment, the cells were thoroughly washed with PBS, fixed with 4% formaldehyde in PBS for 10 min. Cells were then incubated with 10 μg/mL of Hoechst 33342 for 20 min to stain the nuclei at room temperature. The cells were washed again with PBS before the nuclei were visualized using EVOS FL Imaging System (Thermo Fisher Scientific, Waltham, MA, USA).

### 4.6. Measurement of Cellular Oxidation (ROS)

The extent of oxidation was evaluated using CellROX Deep Red Reagent. For 1 h the cells were incubated with CellROX Deep Red Reagent (5 μM) in DMEM in dark followed by rinsing twice with PBS and finally the fluorescence was recorded using fluorescent microscope at the wavelength of 640 nm and 665 nm for excitation and emission respectively. Semi-quantification of ROS level was assessed by using Image J software. All values of %ROS level were normalized to the control group.

### 4.7. Measurement of Mitochondrial Membrane Potential (ΔΨm)

JC-1 dye was used to monitor Δψm. In brief, D407 cells were grown into 96-well plates (2 × 10^4^ cells/well). After treatment, the cells were incubated with JC-1 (10 μg/mL in medium) at 37 °C for 15 min and then washed twice with PBS. For signal quantification, the intensity of red fluorescence (excitation 560 nm, emission 595 nm) and green fluorescence (excitation 485 nm, emission 535 nm) were determined using a Multiskan Ascent Revelation Plate Reader. The ratio of JC-1 red/green fluorescence intensity was calculated for semi-quantitative assessment of mitochondrial polarization states and the value was normalized to the control group.

### 4.8. Caspase 3/7 Activity Assay

The activity of caspase 3/7 was measured using Caspase-Glo^®^ 3/7 Assay kit according to the manufacturer’s protocol. Briefly, D407 cells were lysed in lysis buffer, then centrifuged at 12,500× *g* for 5 min. 15 µL of cell lysate was incubated with 50 µL of 2× substrate working solution at room temperature for 30 min in 96-well plates. The fluorescence intensity was determined by Infinite M200 PRO Multimode Microplate reader at an excitation wavelength of 490 nm and emission wavelength of 520 nm. The fluorescence intensity of each sample was normalized to the protein concentration of sample. All values of % caspase 3/7 activities were normalized to the control group.

### 4.9. Western Blotting

Western blotting was performed as previously described [22]. Briefly, the harvested cells from different experimental conditions were rinsed once with ice-cold PBS and lysed in RIPA buffer. Protein concentration was determined by a BCA protein assay kit according to the manufacturer’s instructions. Aliquots of protein samples (30 µg) were boiled for 10 min at 95 °C and electrophoresed on SDS-PAGE (10% (*w*/*v*) polyacrylamide gel) and then transferred to a nitrocellulose (NC) membrane (Millipore, Billerica, MA, USA). Subsequently, the membranes were blocked with 3% (*w*/*v*) BSA in TBST (TBS containing 0.1% Tween-20) for 1 h at room temperature. The blots were incubated overnight at 4 °C with primary antibodies. After washing with TBST for 30 min at room temperature, the membranes were further incubated with horseradish peroxidase-conjugated secondary antibodies for 1 h at room temperature. Blots were visualized using ECL kit according to the manufacturer’s instructions. The intensity of the bands was semi-quantified using Image J software.

### 4.10. AMPK Silencing by Transfection siAMPK

Before silencing, cells were cultured at 100% confluency. The day before the experiment, the cells were harvested by trypsinization, resuspended in complete medium at the concentration of 1 × 10^5^ cells/mL, and incubated at 37 °C, while the transfection complex was being prepared. Gene silencing of AMPK was performed by specifically synthesized siRNA. On the day of the experiment, transfection siRNA or scrambled sequence as control was incubated with Lipo2000 and opti-MEM following manufacturer’s instructions. After 15 min incubation at room temperature, the obtained complexes were added drop-wise onto the cells sub-cultured in replaced culture medium. The cells were maintained in a 37 °C incubator for 6 h and the medium was replaced with complete medium until analysis. After 48 h from transfection, the cells were collected for protein expression analyses of AMPK. For the MTT assay, cells were seeded into 96-well plates after 24 h from transfection, and MTT assay was performed using standard protocol. The data were quantified by GraphPad Prism 5.0 statistical software.

### 4.11. Statistical Analysis

Statistical analysis was performed using the GraphPad Prism 5.0 statistical software. All experiments were performed in triplicates. Data was expressed as means ± standard deviation (SD). Statistical analysis was carried out using one-way ANOVA followed by Tukey’s multiple comparison, with *p* < 0.05 considered statistically significant.
